# Robust Yet Conductive Blend Anion Exchange Membranes for Hydrogen Production via PPO Reinforcement of Highly Functionalized Styrene–Butadiene-Based Ionomers

**DOI:** 10.3390/membranes16090294

**Published:** 2026-09-02

**Authors:** Andrea Roggi, Marco Turriani, Margherita Di Pede, Gabriele Agonigi, Antonio Filpi, Claudio Resta, Elisa Guazzelli, Elisa Martinelli

**Affiliations:** 1 Department of Chemistry and Industrial Chemistry, University of Pisa, Via G. Moruzzi 13, 56124 Pisa, Italy; andrea.roggi@dcci.unipi.it (A.R.); marco.turriani@ospiti.unipi.it (M.T.); m.dipede5@studenti.unipi.it (M.D.P.); elisa.guazzelli@unipi.it (E.G.); 2 Consorzio Interuniversitario Nazionale per la Scienza e Tecnologia dei Materiali (INSTM), Via G. Giusti 9, 50121 Firenze, Italy; 3 Enapter s.r.l., Via Lavoria, 56040 Crespina Lorenzana, Italy; gagonigi@enapter.com (G.A.); antonio@enapter.com (A.F.); cresta@enapter.com (C.R.)

**Keywords:** poly(phenylene oxide), anionic exchange membranes, water electrolysis, green hydrogen, polymer membranes

## Abstract

Herein, vinylbenzyl chloride (VBC)-grafted styrene-butadiene (SB) copolymers, with high contents of VBC (22–32 mol%), were synthesized and blended with low amounts (*n* = 3–10 wt%) of unfunctionalized poly(phenylene oxide) (PPO). Being well-known for its great chemical compatibility with polystyrene, PPO was selected as non-conductive, hydrophobic and mechanically robust component to be blended with graft copolymers in order to reduce their water uptake, thus improving their dimensional and mechanical stability after quaternization with trimethylamine. The resulting blend membranes were characterized in terms of thermal, mechanical, and *ex situ* electrochemical properties. Blend membranes generally presented improved mechanical properties, as well as reduced water uptake with respect to AEMs not containing PPO, while retaining ion conductivity values of 11.3–16.6 mS cm^−1^, higher than that of a commercial hydrocarbon-based benchmark. Among the investigated blend AEMs, g-VBC-32/PPOn membranes were found to have the highest conductivity values (>15 mS cm^−1^) and the best trade-off between water uptake, mechanical properties and hydrogen permeability. Overall, these results highlight pristine PPO blending as a cost-effective, simple and scalable route to improve the mechanical and dimensional stability of hydrocarbon-based AEMs.

## 1. Introduction

The growing interest in green hydrogen is motivated by the need for alternative energy production pathways that can address some of the most challenging issues regarding industrial decarbonization. Owing to several technological and economic constraints, nowadays steel, cement, glass and the chemical sector still contribute a large portion of unabated emissions [1,2]. In this context, green hydrogen’s pivotal role may not be exclusively limited to fulfilling power production during renewables’ off-peak phases; it could be also actively deployed to reduce the emissions related to all the hydrogen-dependent sectors (e.g., ammonia and methanol production or steel industry) [1,3,4,5].

Water electrolysis has been identified as the technology of choice for hydrogen production thanks to its possibility of being scaled up to industrial level [6]. Low temperature devices can be further divided into conventional alkaline water electrolysis (A-WE), proton exchange membrane water electrolysis (PEM-WE) and the most recent anion exchange membrane water electrolysis (AEM-WE) [6,7]. Although A-WE is widely regarded as the most mature and cost-effective water-electrolysis technology [6,7,8], membrane-based systems, particularly PEM-WE, have enabled more compact cell architectures with reduced ohmic losses, higher operating current densities, and faster dynamic response to load variations. These features make membrane-based electrolysers especially attractive for coupling with intermittent renewable energy sources. However, the acidic environment in PEM-WE limits electrocatalyst choice to a few expensive platinum-group metals (such as Pt, Pd, IrO_2_, and RuO_2_) [6,9,10,11]. Additionally, the excellent cell performance of PEM-WE devices is also due to the use of Nafion™, which, however, is an expensive material that hinders the catalyst recovery due to environmental regulations regarding fluorinated polymeric matrices [10]. These factors lead to high costs and regulatory constraints, which prevent large-scale industrial deployment. On the other hand, the least mature AEM-WE technology combines the advantages of the alkaline environment (cheaper electrocatalysts) with the presence of solid-state electrolytes, which allows for high operating current density (up to 2000 mA cm^−2^) and the adaptability needed to work with discontinuous energy supplies such as renewables [11,12]. Moreover, due to the presence of the polymeric membrane/electrolyte, AEM-WE typically works in milder conditions compared with A-WE, requiring less concentrated electrolyte solutions (KOH or NaHCO_3_ 1–10 wt% for AEM-WE vs. KOH 20–40 wt% for A-WE) and lower operating temperatures (40–60 °C for AEM-WE vs. 70–90 °C for A-WE) [6]. The main limitations of this technology are related to the poorer performance of the AEM, which has to guarantee adequate electrochemical properties and stability under operating conditions, while also being fabricated at low cost [9,13]. Commercial AEMs have costs ranging from 2200 to 7300 $/m^2^ [14] and can achieve durability ranging from 10 to 12,000 h *in operando* AEM-WE cells, still considered insufficient for industrial applications [6]. AEM membranes are often prepared by post-functionalization of commercially available polymers with selected moieties that can be further converted into cationic sites through simple and straightforward procedures. The most employed backbones are based on hydrocarbon polymers (e.g., polyethylene, polystyrene, polynorbornene, styrene-butadiene block copolymers, and polyphenylenes), fluoropolymers (e.g., polytetrafluoroethylene and ethylene-tetrafluoroethylene copolymers) and specialty polymers containing cationic moieties (e.g., polybenzimidazole) [9,11,15,16,17,18]. In this framework, styrene-butadiene (SB) block copolymers are an interesting class of materials that can be easily functionalized by means of simple and straightforward radical grafting [19,20,21,22,23].

Quaternary ammonium groups are the most versatile substrates that can be employed to confer ion exchange capability [9,24,25]. Trimethylammonium, imidazolium and piperidinium are widely investigated in literature and represent the best options to address the trade-off among electrochemical performance, cost and chemical stability [9,26,27]. Although a high concentration of ionic sites is usually associated with an improved electrochemical performance, this may also result in larger amounts of incorporated water and a consequent loss of mechanical robustness [28,29].

Mechanical reinforcement represents an interesting strategy to obtain robust and dimensionally stable AEMs under operating conditions, even with a high concentration of ionic sites in the ion exchange ionomer [30,31,32,33]. It is generally achieved by means of different methods that include chemical and physical cross-linking [18,34,35], impregnation of a robust, a non-conducting matrix [28,29,36] with ion exchange ionomers and the incorporation of additives (both organic and inorganic species) [37,38].

From an industrial point of view, physical polymer blending is extremely attractive, being an easy and cost-effective approach for the development of novel polymeric materials with improved properties with respect to the pristine components. In the field of AEMs, it is emerging as an effective strategy to improve the balance between ionic conductivity, dimensional stability, and mechanical robustness. For example, side-chain-functionalized polystyrene/O-PBI blends exhibited OH^−^ conductivities up to 54 mS cm^−1^ at 80 °C and showed no measurable conductivity loss after six weeks in 1 M KOH at 85 °C, while achieving 2.0 A cm^−2^ at below 1.8 V in an AEM water electrolyzer [39]. Other PBI-based blends with poly(vinylbenzyl chloride) [40], polyarylimidazoliums [41], quaternized poly(etherether ketone) (QPEEK) [42], and poly(oxindole biphenylene) [43] also demonstrated that suitable polymer–polymer interactions can simultaneously improve conductivity, mechanical properties, and gas-barrier behavior.

However, the success of this approach is strictly related to the degree of miscibility among the involved polymeric components. Indeed, only completely miscible blends or at least partially miscible blends, which originate micro-phase separation at the nanoscale, generally led to an improvement of the properties of the material with respect to the pristine polymeric components [44,45,46].

Poly(*p*-phenylene oxide) (PPO) is an engineering polymer with good resistance in aggressive chemical environments; it is particularly attractive for blending because of its excellent mechanical and thermal properties and its well-established compatibility with polystyrene-rich phases [47,48,49]. Thanks to such properties, PPO has been investigated in recent years as a starting material for the preparation of AEMs after the functionalization for the incorporation of ionic sites [50,51,52,53]. Also, there are a few examples of the use of quaternized PPO in blend AEMs [54,55,56,57,58,59], including commercial products such as FAA-3 by Fumatech [60]. For instance, partially crosslinked quaternized PPO membranes reached ionic conductivities of 133 mS cm^−1^ at 80 °C and retained up to 94% of their initial IEC after 500 h in 1 M KOH [50], while optimized PPO-containing blends showed conductivities up to 90.9 mS cm^−1^ together with controlled swelling and prolonged alkaline stability [55]. Earlier chloroacetylated PPO/bromomethylated PPO (CPPO/BPPO) blends for alkaline direct methanol fuel cells also achieved OH^−^ conductivities of 22–32 mS cm^−1^ at 25 °C, with crosslinking further improving their mechanical and barrier properties [57,58]. Importantly, in these PPO-containing AEMs, PPO is chemically functionalized and participates directly in ion transport, intermolecular interactions and/or crosslinking. Otherwise, to the best of our knowledge the use of non-functionalized PPO as a physically blended reinforcing component of thermoplastic elastomer-based AEMs has not been previously explored. This approach addresses a central challenge in AEM design: improving mechanical and dimensional stability without introducing additional synthetic complexity or severely compromising ionic conductivity.

By taking advantage of the high miscibility of PPO with polystyrene, in this work we report the use of non-functionalized PPO as a reinforcing filler of vinylbenzyl chloride (VBC) graft styrene-butadiene copolymers (g-VBC-x)-based AEMs, which were found to have high water uptake values resulting in unsuitable mechanical properties for the envisaged application. Indeed, it is expected that the blending of small amounts of non-functionalized PPO with highly functionalized g-VBC-x copolymers reduces excessive water uptake and improves mechanical and dimensional stability, while preserving the sufficiently high ionic conductivity of the membranes obtained after quaternization for use in AEM-WE. Accordingly, this work addresses three main aspects: (i) how does PPO loading affect the hydration and mechanical properties of highly functionalized g-VBC-based AEMs; (ii) what is the impact of PPO incorporation on ion conductivity and hydrogen permeability; and (iii) how the combination of VBC functionalization degree and PPO content provides the most favorable performances. Therefore, starting from these rationales, we synthesized g-VBC-x copolymers, with a high content of VBC, i.e., functionalization degree (FD or x = 22–32 mol%), and used them for the preparation of blends with different amounts of PPO (3–10 wt%). The prepared films were aminated with trimethylamine (TMA) and converted into blend AEMs, which were extensively characterized to assess their thermal, mechanical and ex-situ electrochemical properties and compared with the corresponding AEMs not containing PPO. There was a particular focus on the identification of the best balance between VBC FD of graft copolymers and PPO content in the blends to address the trade-off between water uptake, ionic conductivity and mechanical performance, with the final aim being the obtainment of more robust yet highly conductive AEMs. Although aryl–ether-containing polymers can undergo degradation under harsh alkaline conditions, particularly at elevated temperatures and low hydration levels, this concern is especially relevant for aryl–ether backbones containing strongly electron-withdrawing groups. Moreover, the present blend AEMs containing only a small amount of non-functionalized PPO are intended to operate under comparatively mild conditions, namely, in water or dilute KOH solution (1 wt%) at 50–55 °C [22].

## 2. Materials and Methods

### 2.1. Materials

Methanol, HPLC-chloroform (ethanol-stabilized) and chloroform were purchased from Carlo Erba (Cornaredo (MI), Italy) and used without further purification. Benzoyl peroxide (BPO) was purchased from Carlo-Erba (Cornaredo (MI), Italy) and recrystallized from chloroform/methanol. Vinylbenzyl chloride (VBC) (97% purity; a mixture of 2-, 3-, and 4-isomers) was purchased from Sigma Aldrich (Darmstadt, Germany) and purified via repeating washings with 5% NaOH solutions and water to remove inhibitors. After drying over Na_2_SO_4_, VBC was distilled under reduced pressure. Hydroquinone monomethyl ether (97% purity) was purchased from Sigma Aldrich and used without further purification. Trimethylamine aqueous solution (TMA, 45% wt/v), acetone and deuterated solvents were purchased from Sigma Aldrich (Darmstadt, Germany) and used without further purification. A poly(styrene-*b*-butadiene) (SB) block copolymer was kindly provided by Enapter (Crespina Lorenzana, Italy). After purification by two precipitations into methanol from chloroform solutions, SB copolymer was found to be composed of 36 mol% of butadiene (33 mol% and 3 mol% of 1,4 and 1,2 isomers, respectively) and have a number average molecular weight (*Mn*) and dispersity (*Ð*) of 89 800 g mol^−1^ and 1.40, respectively.

### 2.2. Preparation of Blend AEM

Poly(vinylbenzyl chloride) grafted poly(styrene-b-butadiene) copolymers (g-VBC-x) with x = 22–32 mol% VBC were prepared according to a previously reported and optimized procedure [22] briefly described in the Supporting Information (SI). Solution casting of g-VBC-x/PPOn blends and quaternization of the films were carried out by following the procedure reported in our previous work [22]. As a typical example, copolymer g-VBC-32 (1540 mg) and PPO (46 mg) were dissolved in chloroform (100 mL) at room temperature. Then, a chloroform solution of hydroquinone monomethyl ether (0.86 mL, 0.125 mg mL^−1^) was added under stirring. After that, the mixture was filtered on a filter paper, sonicated for 10 min and poured in a glass Petri dish, which was left overnight in a chloroform saturated chamber under the fume hood to slowly evaporate the solvent. The thickness of the dry films was 70–100 μm. The obtained film containing 3 wt% PPO was named g-VBC-32/PPO3. Later, TMA was added to an Erlenmeyer flask containing methanol to obtain a final concentration of 2.5% wt/v and g-VBC-x/PPOn films were immersed in the solution and left for 5 days at room temperature. Then, the films were repeatedly washed with deionized water and air-dried at room temperature.

### 2.3. Characterization

^1^H-NMR spectra of g-VBC-x copolymers were recorded with a JEOL CZR 500 MHz and JEOL YH400 MHz spectrometers (JEOL Ltd., Akishima, Tokyo, Japan). Number- and weight-average molecular weights of g-VBC-x copolymers were determined by gel permeation chromatography (GPC) using a Jasco (JASCO Corporation, Hachioji-shi, Tokyo, Japan) PU-2089Plus liquid chromatograph equipped with two PL gel 5 µm mixed-D-columns, a Jasco RI-2031Plus refractive index detector and a Jasco UV-2077Plus UV/vis detector (JASCO Corporation, Tokyo, Japan). Polystyrene standards were used for calibration (400–400,000 g mol^−1^). FT-IR spectra of AEMs were recorded on a Perkin-Elmer (PerkinElmer, Waltham, MA, USA) Spectrum One spectrometer. Differential scanning calorimetry (DSC) analysis was performed with a Mettler (Mettler Toledo, Columbus, OH, USA) DSC 922e calorimeter. Samples were dried under vacuum at room temperature and stored in a dry environment before measurement. Thermograms were recorded starting from −120 °C to 260 °C with a heating rate of 10 °C min^−1^. The glass transition temperatures (*T*_g_) were determined as the inflection point of the curve in the second heating cycle. Thermogravimetric analysis was performed using a thermogravimetric analyzer Linseis (Linseis Messgeraete GmbH, Selb, Germany) TGA PT 1000 under nitrogen inert flux by heating from 30 °C to 700 °C at 10 °C min^−1^. The mechanical properties of membranes were evaluated by stress-strain tests. Dogbone-shaped specimens (21.1 mm × 4.75 mm) were cut and conditioned overnight in 1 M NaHCO_3_ solution at room temperature. The specimens were swabbed with filter paper. The stress-strain properties at a grip separation rate of 10.5 mm min^−1^ of the sample were recorded with a Tinius Olsen instrument (Tinius Olsen, Horsham, PA, USA). Water uptake (WU) was determined through a gravimetric method described in SI. The ion exchange capacity (IEC) of the membrane was measured by acid-base back titration described in SI. Ionic conductivity measurements and hydrogen permeability through the membranes were determined through a potentiostat/galvanometer characterized by an impedance channel equipped with a 4 A AC generator (VMP3 Bio-Logic, Thane, MA, USA) and a cell hardware (Fuel Cell Technologies Inc., Albuquerque, NM, USA). The methods used are described in the SI.

## 3. Results and Discussion

### 3.1. Membrane Preparation

**Figure 1:**
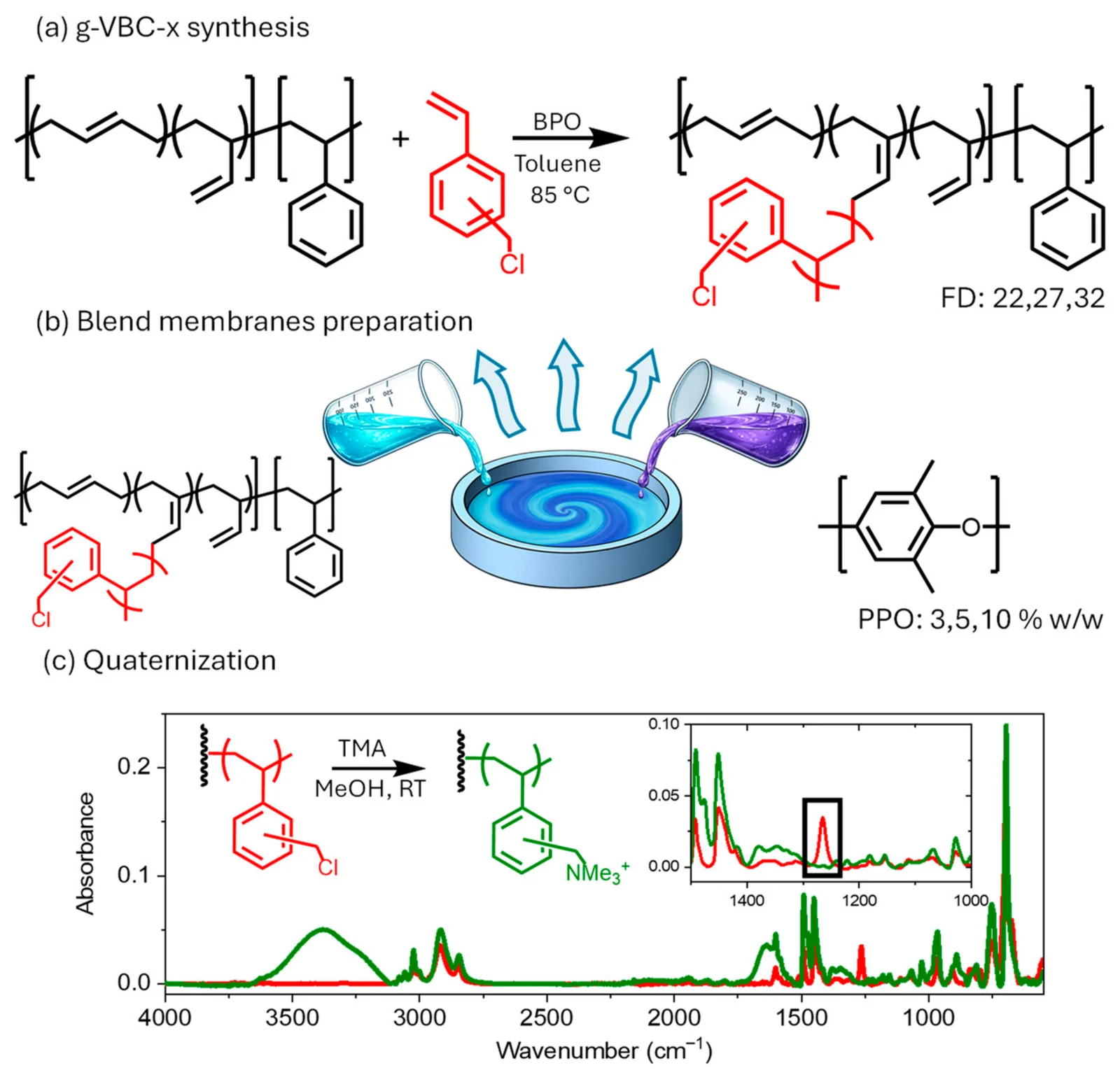
Steps for the preparation of blend AEMs: Scheme of the synthesis of g-VBC-x copolymers (**a**). Blend film preparation through solution casting (**b**). Quaternization of blend AEMs. The reaction was followed through FT-IR, in particular by monitoring the disappearance of the signal at 1265 cm^−1^ relative to the C-Cl wagging (**c**). In red the FT-IR spectra before the quaternization and in green the spectra after the quaternization.

A commercial styrene-butadiene copolymer was modified by grafting VBC units using the procedure developed and optimized in previous studies [22]. Briefly, the functionalization was carried out at 85 °C in a toluene solution, with benzoyl peroxide (BPO) as a radical initiator (Figure 1a). By varying the reaction conditions (i.e., reaction time and the VBC/SB ratio), it was possible to tune the amount of grafted VBC in the final copolymer, i.e., the functionalization degree (FD or *x*). The copolymer composition was determined by ^1^H NMR (Figure S1). For this work, the g-VBC-graft copolymers were designed to have a medium/high FD, which was in the range 22–32 mol%.

Homogeneous polymeric films were prepared through solution casting of mixtures obtained by dissolving increasing amounts of PPO into a chloroform solution of g-VBC-x (~13 mg mL^−1^) to prepare blends with specific PPO contents (3–10 wt%). Chloroform was selected as a suitable solvent for both g-VBC copolymers and PPO, while also allowing easy removal by evaporation. All the obtained films were transparent without visible surface imperfections and thickness between 70 μm and 100 μm. The prepared films were named as g-VBC-x/PPOn, where x is the functionalization degree, i.e., the mole percentage of VBC in the copolymer, and *n* the weight percentage of PPO in the blend (Figure 1b). The type of g-VBC-x copolymer and PPO quantities used for film preparation are reported in Table 1.

The VBC moieties were then converted into ionic sites by reaction with TMA. Amination reaction was carried out after blending, because the polarity and solubility of the g-VBC copolymers change significantly after quaternization. Indeed, the solubility of g-VBC-based ionomers in chloroform became very limited as a result of their increased polarity and hydrophilicity. The quaternization reaction was followed by FT-IR spectroscopy until the disappearance of the peak at 1265 cm^−1^ (attributed to CH_2_Cl groups), thus indicating the complete conversion of the CH_2_Cl moieties of the VBC units into quaternary ammonium cationic groups (Figure 1c).

### 3.2. Thermal Characterization of Blend AEMs

**Figure 2:**
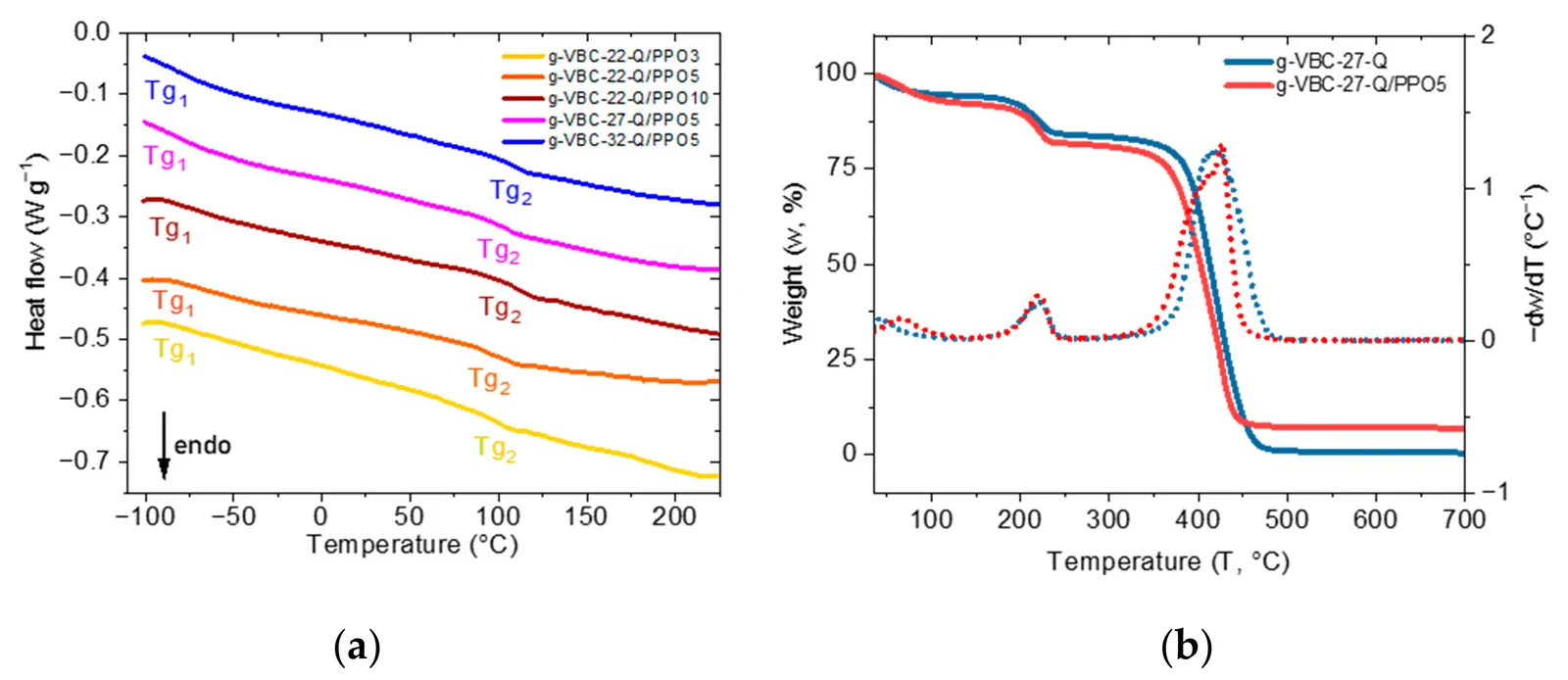
DSC traces (**a**) and TGA curves (**b**) for blend AEMs. Solid lines: weight (%); dashed lines: first-order derivative of weight, multiplied by −1.

The thermal behavior of g-VBC-x-Q/PPOn blend membranes was investigated by differential scanning calorimetry (DSC) and thermogravimetric analysis (TGA) and compared with those of the membranes derived from the corresponding pristine graft copolymer. All the g-VBC-x-Q/PPOn blend membranes exhibited two glass transitions (Figure 2a) in the second heating cycle in the range −86/−74 °C for *T*_g1_ and 102/110 °C for *T*_g2_, due to the polybutadiene-rich soft phase and polystyrene-rich hard phase. Both *T*_g1_ and *T*_g2_ were consistent and comparable with those presented by the membranes derived from the corresponding pristine graft copolymers (Table S2). There was no clear evidence of the presence of a transition associated with the PPO component, indicating that, as expected, it was incorporated in a miscible blend with the polystyrene backbone of the main and grafted chains. The results therefore confirmed the presence of a phase-separated morphology at the nanoscale in the blend AEMs, which was similar for both membranes with and without PPO.

TGA analysis of blend membranes showed two degradation steps (Figure 2b, Table S3); the first one at around 220 °C was attributed to the elimination of the quaternary ammonium moieties, while the second one at around 425 °C was possibly related to the polymer matrix composed of the graft copolymer backbone and PPO. Such thermal degradation behavior was in line with that presented by the corresponding g-VBC-x-Q-based membranes, thus indicating that the addition of relatively low amounts of PPO did not significantly affect the thermal behavior of the blend membranes. However, blend membranes presented a higher residue at 700 °C (7–15 wt%) with respect to the pristine graft copolymer, consistent with the formation of char as expected for the degradation of PPO [61].

### 3.3. Water Uptake and Ex-Situ Electrochemical Properties

Water uptake (WU), ion exchange capacity (IEC), ion conductivity *σ*_TP_ and hydrogen permeability (H_2_ P) for the blend AEM and the corresponding g-VBC-x-based ones are collected in (Table 2). In general, WU plays a central role in AEM for water electrolysis in determining the balance between ionic conductivity and mechanical stability. On the one hand, sufficient water absorption is essential to hydrate and promote the interconnection of ionic domains for efficient hydroxide ion transport; on the other hand, excessive WU induces pronounced swelling, which reduces dimensional and mechanical stability. In this work, AEMs deriving from g-VBC-x graft copolymers with relatively high VBC FD (22–32 mol%) displayed large WU values ≥ 131%, much higher than the typical value (WU = 44%) of a commercially available benchmark (Table 2). With the aim to reduce their swelling and improve mechanical stability, PPO-based blend membranes were investigated, with an additional focus on the effect of PPO on electrochemical properties. Indeed, g-VBC-x/PPOn blend membranes were found to show reduced WU, which tended to decrease with increasing PPO content in the blend. For example, in g-VBC-22-Q and g-VBC-27-Q, the addition of 5 wt% PPO led to a reduction in WU of about 30%, whereas for g-VBC-32-Q the same amount of PPO resulted in a decrease of 16%. Similarly, IEC values of blend membranes were generally lower than those of the corresponding ones without PPO, apart from the series with the highest FD (g-VBC-32), in which the IEC remained mostly unchanged. These trends are consistent with the incorporation of a non-ion conductive hydrophobic polymer, such as PPO, that led to a dilution of the cationic groups resulting in a reduction in WU and IEC. Electrochemical tests indicated that the inclusion of PPO led to a decrease in *σ*_TP_, as a result of the reduction in IEC. This occurred for any FD investigated at any given content of PPO. For example, g-VBC-22-based membranes displayed *σ*_TP_ values that decreased from 16.8 mS cm^−1^ for no-PPO membranes to 12.1 mS cm^−1^, and even down to 4.7 mS cm^−1^ for PPO contents of 3 wt% and 10 wt%, respectively. This was still consistent with the non-ionic conductive behavior of PPO. The *σ*_TP_ value obtained for the blend containing 10 wt% PPO was considered too low for practical applications, and, therefore, a maximum content of 5 wt% PPO was selected for PPO-based AEMs. For a given value of PPO weight percentage, *σ*_TP_ increased by increasing the FD of the copolymer. The blend membrane with the highest *σ*_TP_, equal to 16.6 mS cm^−1^, was g-VBC-32-Q/PPO3, having the highest FD and the lowest amount of PPO.

Hydrogen permeability is another critical parameter to evaluate in operating electrolytic cells. In particular, limiting hydrogen transport through the polymeric membrane from the cathode to the anode is essential for two main reasons: first, to minimize safety risks associated with the possible formation of explosive hydrogen/oxygen mixtures under operating conditions, and second, to preserve cell efficiency, since increased hydrogen permeability results in greater losses in hydrogen production. The values measured for the blend AEMs showed a slight decrease in hydrogen permeability, by the addition of PPO in the blend, especially for the g-VBC-22-Q and g-VBC-32-Q-based series. Interestingly, the g-VBC-32-Q/PPO3 blend membrane, which showed one of the highest *σ*_TP_ values, also exhibited the lowest hydrogen permeability among the investigated blends. Although this value was higher than those reported for the commercial FAA-3 and Tokuyama A201 membranes under alkaline conditions at 50 °C (7.3 × 10^−17^ and 5.3 × 10^−17^ mol cm^−1^ s^−1^ Pa^−1^, respectively), it can still be considered acceptable for the envisaged application [13].

### 3.4. Mechanical Characterization

**Figure 3:**
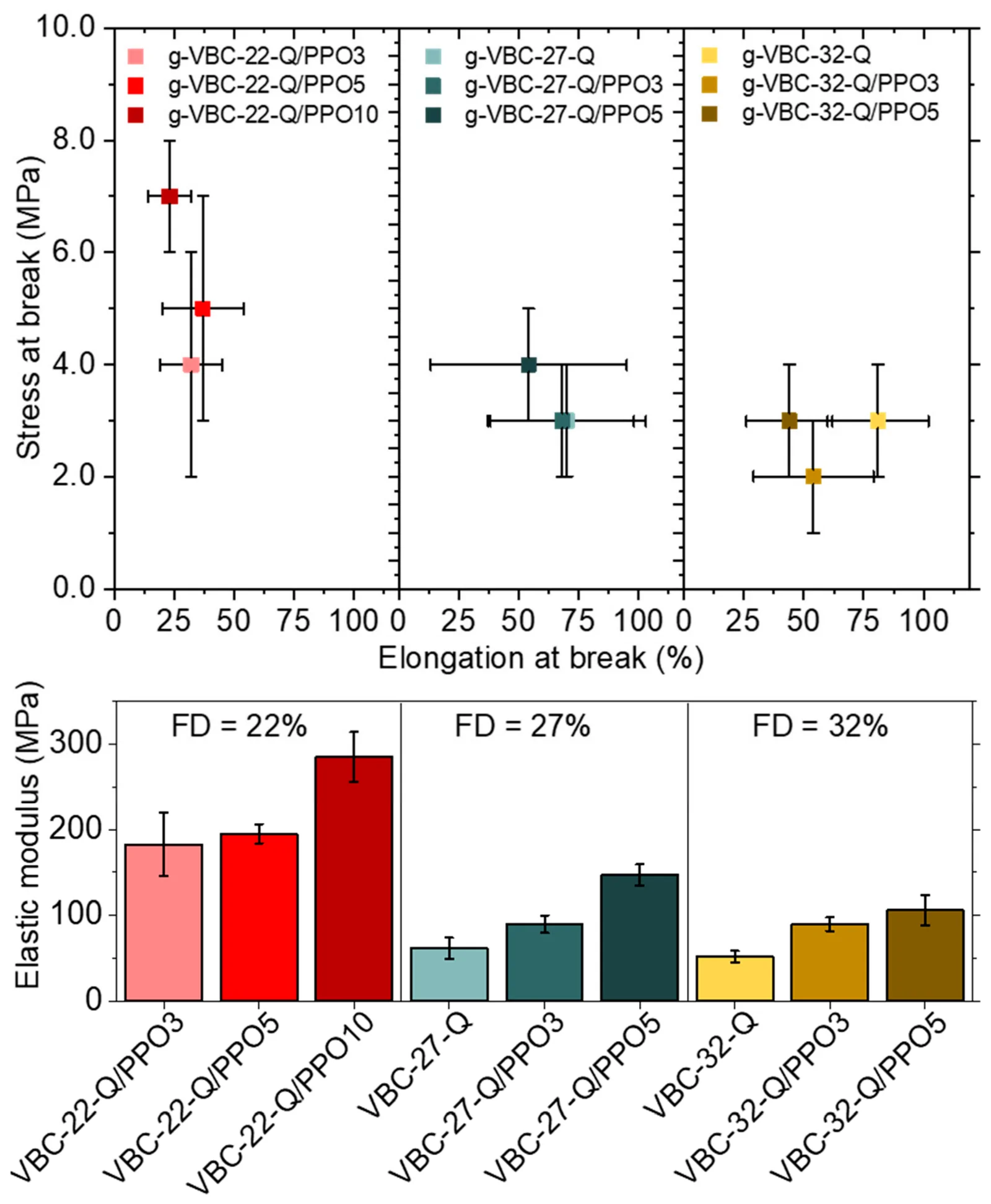
Tensile strength (*σ*_max_), elongation at break (*ε*_b_) and elastic modulus (*E*) of g-VBC-x-Q/PPOn and the corresponding g-VBC-x-Q membranes.

Tensile strength (σ_max_), elongation at break (ε_b_) and Young’s modulus (E) of g-VBC-x-Q/PPOn and the corresponding g-VBC-x-Q membranes, after being conditioned overnight in 1 M KHCO_3_ aqueous solution, are reported in Table S4. The mechanical performance of g-VBC-x-Q/PPOn membranes was significantly improved, especially in terms of Young’s modulus, with respect to those of the corresponding pristine g-VBC-x-Q AEMs. Moreover, the elastic modulus tended to increase by increasing the content of PPO in the blend and decrease with the FD, for a given PPO content. Elongation at break showed the opposite trend, thus decreasing with PPO content because of its high rigidity. As an example, for g-VBC-27-based membranes E increased from 60 MPa, to 90 MPa, up to 147 MPa by increasing the PPO content from 0 wt%, to 3 wt%, up to 5 wt% (Figure 3). On the other hand, the elongation at break decreased from 70% for g-VBC-27-Q to 54% for g-VBC-27-Q/PPO5. The tensile stress tended to slightly increase with PPO (Figure 3). Overall, these results demonstrate an improvement in mechanical performance upon PPO addition, particularly in terms of elastic modulus. Although the absolute tensile-strength values remain modest, both the tensile strength and elastic modulus are consistent with the elastomeric nature of the SB-based starting material. Moreover, in a previous work, analogous VBC-based AEMs with comparable tensile strength (~3 MPa) and lower elastic modulus (~62 MPa) were successfully assembled into a single-cell electrolyzer and demonstrated stable long-term AEM-WE operation under mild conditions (1 wt% KOH, corresponding to 0.178 M, at 55 °C) [22].

## 4. Conclusions

This work demonstrates that small amounts of pristine PPO (3–10 wt%) can be effectively used as a non-conductive reinforcing component for highly functionalized g-VBC-x ionomers (x = 22–32 mol%) to produce mechanically strengthened blend AEMs. PPO incorporation led to improved mechanical stiffness, while determining a decrease in water uptake and ionic conductivity. The extent of these changes increased with PPO loading, highlighting the need to balance water uptake, ion transport and mechanical reinforcement. Among the investigated formulations, g-VBC-32-Q/PPOn membranes offered the most favorable compromise, keeping through-plane conductivity above 15 mS cm^−1^ while showing reduced water uptake, significantly enhanced elastic modulus and acceptable hydrogen permeability. These properties make them promising candidates for validation in AEM-WE cell tests. Overall, pristine PPO blending emerges as a straightforward and scalable strategy to shift the membrane properties toward a more favorable balance between water uptake, conductivity and mechanical robustness. This balance is particularly relevant for highly functionalized hydrocarbon AEMs, where excessive hydration often compromises dimensional and mechanical stability as well as hydrogen permeability.

## Figures and Tables

**Table 1:** Type of g-VBC-x copolymer and PPO quantities used for film preparation.

Film	VBC FD (mol%)	g-VBC-x (g)	PPO (g)	PPO (wt%)
g-VBC-22/PPO3	22	1.908	0.059	3
g-VBC-22/PPO5	22	1.596	0.084	5
g-VBC-22/PPO10	22	1.548	0.172	10
g-VBC-27/PPO3	27	1.487	0.046	3
g-VBC-27/PPO5	27	1.463	0.077	5
g-VBC-32/PPO3	32	1.487	0.046	3
g-VBC-32/PPO5	32	1.463	0.077	5

**Table 2:** Water uptake (WU), ion exchange capacity (IEC), ion conductivity *σ*_TP_ and hydrogen permeability (H_2_ P) for the g-VBC-x AEMs and the g-VBC-x/PPO blend AEMs.

Membrane	H_2_ P (mol cm^−1^ s^−1^ Pa^−1^)	*σ*_TP_ ^a^ (mS cm^−1^)	IEC ^b^ (mmol g^−1^)	WU ^b^ (%)
g-VBC-22-Q	9.6 × 10^−17^	16.8	1.9 ± 0.1	159 ± 6
g-VBC-22-Q/PPO3	14.7 × 10^−17^	12.1	0.9 ± 0.1	121 ± 1
g-VBC-22-Q/PPO5	11.5 × 10^−17^	11.3	1.0 ± 0.1	109 ± 1
g-VBC-22-Q/PPO10	10.2 × 10^−17^	4.7	1.2 ± 0.1	112 ± 8
g-VBC-27-Q	12.1 × 10^−17^	19.5	2.1 ± 0.3	177 ± 1
g-VBC-27-Q/PPO3	13.0 × 10^−17^	14.4	1.5 ± 0.2	167 ± 21
g-VBC-27-Q/PPO5	11.7 × 10^−17^	14.9	1.4 ± 0.1	130 ± 5
g-VBC-32-Q	13.6 × 10^−17^	18.6	1.3 ± 0.1	131 ± 6
g-VBC-32-Q/PPO3	10.4 × 10^−17^	16.6	1.4 ± 0.1	134 ± 11
g-VBC-32-Q/PPO5	10.9 × 10^−17^	15.2	1.3 ± 0.1	110 ± 13
Benchmark ^c^	-	7.3	1.5 ± 0.3	44 ± 5

^a^ Through-plane conductivity at 25 °C. A 1 M KHCO_3_ solution was fed for all the duration of the test. ^b^ Films were conditioned for 12 h in 1 M KOH aqueous solution before being tested. ^c^ Literature data reported in [62].

## Data Availability

The data that support the findings of this study are available from the corresponding author upon reasonable request.

## Supplementary Materials

The following supporting information can be downloaded at https://www.mdpi.com/article/10.3390/membranes16090294/s1, Table S1: Experimental parameters for the synthesis of g-VBC-x copolymers; Figure S1: ^1^H NMR spectra in CDCl_3_ of g-VBC-22; Table S2: Glass transition temperatures (T_gs_) and heat capacity variations (ΔC_p_) for g-VBC-x-Q/PPOn and the corresponding g-VBC-x-Q membranes; Table S3: Temperatures of maximum degradation (T_n_), weight loss (Δw_n_), and residue for g-VBC-x-Q/PPOn and the corresponding g-VBC-x-Q membranes; Table S4: Young’s modulus (E), tensile strength (σ_max_) and elongation at break (ε_b_) of g-VBC-x-Q/PPOn membranes and relative g-VBC-x-Q copolymers. Films were conditioned overnight in 1 M KHCO_3_ aqueous solution before being tested.
