# Supplementary material for: Robust Yet Conductive Blend Anion Exchange Membranes for Hydrogen Production via PPO Reinforcement of Highly Functionalized Styrene–Butadiene-Based Ionomers
Source: Membranes (Basel). 2026 Sep 2;16(9):294. doi: 10.3390/membranes16090294 (PMC13609407; doi:10.3390/membranes16090294)
Supplement: Supplementary file 1 [file membranes-16-00294-s001.zip › membranes-4513087-supplementary.pdf]

Robust Yet Conductive Blend Anion Exchange Membranes for Hydrogen Production via PPO Reinforcement of Highly Functionalized Styrene–Butadiene-Based Ionomers

Andrea Roggi [1], Marco Turriani [1,2], Margherita Di Pede [1], Gabriele Agonigi [3], Antonio Filpi [3], Claudio Resta [3], Elisa Guazzelli [1] and Elisa Martinelli [1,*]

[1] Department of Chemistry and Industrial Chemistry, University of Pisa, Via G. Moruzzi 13, 56124 Pisa, Italy; andrea.roggi@dcci.unipi.it (A.R.); marco.turriani@ospiti.unipi.it (M.T.); m.dipede5@studenti.unipi.it (M.D.P.); elisa.guazzelli@unipi.it (E.G.)

[2] Consorzio Interuniversitario Nazionale per la Scienza e Tecnologia dei Materiali (INSTM), Via G. Giusti 9, 50121 Firenze, Italy

[3] Enapter s.r.l., Via Lavoria, 56040 Crespina Lorenzana, Italy; gagonigi@enapter.com (G.A.); antonio@enapter.com (A.F.); cresta@enapter.com (C.R.)

* Correspondence: elisa.martinelli@unipi.it

SUPPORTING INFORMATION

Recrystallization of benzoyl peroxide

Commercial benzoyl peroxide was purified by recrystallization from methanol. BPO was suspended in methanol and the mixture was warmed at 50°C, using the minimum amount of solvent required to obtain a homogeneous solution. The solution was allowed to cool gradually to room temperature and was subsequently cooled further (0°C, overnight) to promote crystallization. The resulting colorless crystals were collected by filtration and washed with a small amount of cold methanol. The purified BPO was stored at 0°C.

Purification of vinylbenzyl chloride

Vinylbenzyl chloride (VBC, mixture of 2-, 3-, and 4-isomers) was purified prior to use to remove the polymerization inhibitor. The monomer was transferred to a separatory funnel and washed three times with an equal volume of 5 wt% aqueous NaOH. After each extraction, the phases were allowed to separate and the aqueous phase was discarded. The organic phase was subsequently washed several times with deionized water until the aqueous washings were neutral. The purified VBC was then dried over anhydrous Na_2SO_4, filtered to remove the drying agent, and distilled under reduced pressure (~ 60°C at < 1mmHg). The purified monomer was collected and stored under an inert atmosphere at 20°C until use.

Purification of poly(styrene-*b*-butadiene) block copolymer

The poly(styrene-*b*-butadiene) (SB) block copolymer, was purified prior to use by two successive precipitations from chloroform into methanol. The copolymer was completely dissolved in chloroform at room temperature, and the resulting solution was slowly added dropwise to an excess of methanol (1:10 vol:vol) under stirring to induce precipitation. The precipitated polymer was collected, dried and redissolved in fresh chloroform. The precipitation procedure was then repeated a second time using fresh methanol. After the second precipitation, the purified polymer was recovered and dried under reduced pressure at room temperature until constant mass.

Synthesis of g-VBC-x copolymers

g-VBC-x copolymers were synthesized according to a previously optimized procedure[21]. SB copolymer (4 g, 0.045 mmol) and BPO (0.14 g, 0.83 mmol) were added to a 250 mL three-necked round-bottom flask equipped with a magnetic stirrer and a condenser. The oxygen was removed by nitrogen/vacuum cycles and the reactants were dissolved in toluene (28 mL) and VBC (11.5 mL, 815 mmol) at room

temperature. Once the solids were completely dissolved, the temperature was increased up to 85 °C. The reaction was carried out for 210 minutes under inert atmosphere. The product was purified by a precipitation into a 280 mL acetone/methanol (70/30 v/v) mixture from the reaction solution. The obtained dispersion was centrifuged and the white solid dried and dissolved again in 40 mL of chloroform for a further precipitation in 400 mL of methanol. A white product (g-VBC-32) was recovered and dried under vacuum at room temperature (M_n = 59000 g mol^{-1}; $Đ$ = 1.99). The purified graft copolymer, containing 32 mol% VBC, was named g-VBC-32.

Table S1. Experimental parameters for the synthesis of g-VBC-x copolymers

**Graft copolymer**	**VBC mol%**[a]	**BPO/SB wt/wt**	**VBC/SB wt/wt**	**Time (min)**	***M*** M_n[b] **(g/mol)**	***Đ***[b]
g-VBC-22	22	0.02	2.38	130	68000	1.74
g-VBC-27	27	0.02	3.57	210	78200	1.67
g-VBC-32	32	0.02	3.11	210	59000	1.99

[a]Functionalization degree (FD) [b]Number average molecular weight and dispersity of the copolymer determined by GPC

^{1}H NMR

^{1}H, $CDCl_3$: 7,2-6,3 ppm (arom. styrene and arom. VBC), 5,6 ppm ($-C\underline{H}=CH_2$ 1,2 but.), 5,4 ppm ($-C\underline{H}=C\underline{H}-$ 1,4 but.), 5,0 ppm ($-CH=C\underline{H_2}$ 1,2 but.), 4,6-4,3 ppm ($-Ph-C\underline{H_2}-Cl$), 2,5-1,2 ppm (aliphatic protons).

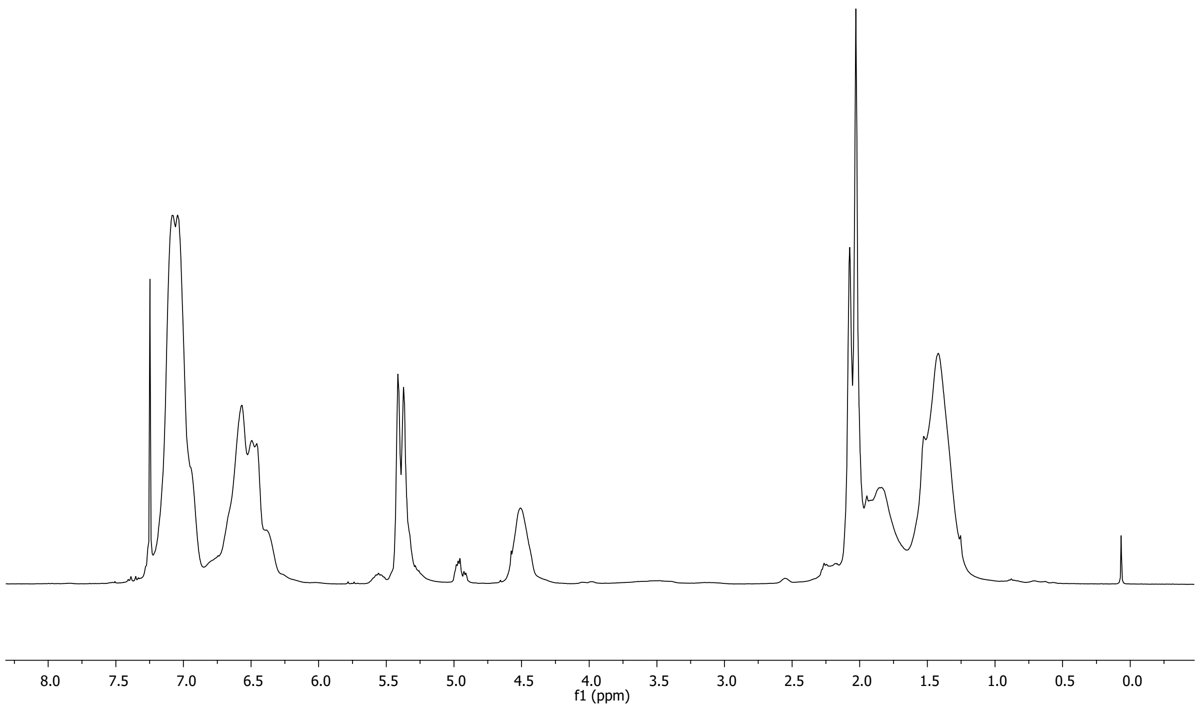

Figure S1. ^{1}H NMR spectra in $CDCl_3$ of g-VBC-22

Membrane characterization

Water uptake

Water uptake (WU) was determined according to the following procedure. The dry weight (w_{dry}) of the membrane in Cl^- form was measured after drying overnight under vacuum at room temperature. The membrane was then conditioned in 1 M KOH aqueous solution for about 12 hours at room temperature. Wet weight (w_{wet}) was measured after rinsing the sample with deionized water to remove the excess of adsorbed KOH and wiped with humidified filter paper. Water uptake was calculated using Equation 1:

$$WU_{KOH}(\%) = \frac{W_{KOH} - W_{Dry}}{W_{Dry}} \cdot 100 \qquad \text{Eq. 1}$$

Ion exchange capacity

Ion exchange capacity (IEC) of the membrane was measured by acid-base back titration. Specifically, the membrane was previously cut into pieces and dried under vacuum at room temperature for 24 hours. The dried stripes were weighed in a closed vessel and then immersed in 1 M KOH and left overnight at room temperature. Samples were rinsed thoroughly with deionized water and immersed in 25 mL 0.01 N HCl standard solution for 2 h. After that, samples were taken away and the solutions were diluted to 100 mL with deionized water. The solutions were conductometrically titrated with 0.01 N NaOH standard solution. Finally, samples were dried in an oven at 50 °C overnight. The IEC was calculated according to Equation 2:

$$\mathrm{IEC}\ (\mathrm{mmol} \cdot \mathrm{g}^{-1}) = \frac{V_{HCl} \cdot c_{HCl} - V_{NaOH}^{eq} \cdot c_{NaOH}}{m_{dry}} \qquad \text{Eq. 2}$$

where V_{HCl} is the starting volume of HCl solution, c_{HCl} is HCl solution concentration, V_{NaOH}^{eq} is NaOH solution volume at the equivalent point, c_{NaOH} is NaOH solution concentration and m_{dry} is the weight of the dry sample.

Ionic conductivity

Ionic conductivity measurements were conducted through a potentiostat/galvanometer characterized by an impedance channel equipped with a 4 A AC generator (VMP3 Bio-Logic). The membrane was assembled between two electrodes of commercial catalyst Pt/C, containing about 0.2 $mg \cdot cm^{-2}$ of platinum. Then, the MEA was put in a cell hardware having an active area of 5 cm^2 (Fuel Cell Technologies inc.). The cell was connected to a peristaltic pump that ensured a continuous flow of 1 M $KHCO_3$ aqueous solution. Before the measurements, the cell was conditioned for 20 min at constant temperature (R.T.) and flow. The impedance due to the membrane was measured by imposing a sinusoidal AC voltage of 5 mV vs Open Circuit Voltage (OCV) at frequency ranging from 100 kHz to 100 Hz into the cell. Ionic conductivity was then calculated as follow[21] (Equation 3):

$$\sigma\ (\mathrm{mS} \cdot \mathrm{cm}^{-1}) = \frac{d\ (\mathrm{cm})}{\mathrm{HFR}\ (\Omega) \cdot A\ (\mathrm{cm}^2)} \cdot 1000 \qquad \text{Eq. 3}$$

where HFR (High Frequency Resistance) is the resistance obtained by fitting high frequency impedance data on a Nyquist plot to a 45° transmission line, *A* is the electrode area and *d* is the membrane thickness. The electrical resistances of the plates, Gas Diffusion Electrodes (GDEs) and interfaces were considered as negligible.

Hydrogen permeability

Hydrogen permeability through the membrane was determined with electrochemical measurements. A membrane electrode assembly (MEA) was assembled by placing the membrane, previously conditioned in 1 M KOH, between 2 electrodes containing approximately 0.2 mg cm^{-2} of platinum using a commercial Pt/C catalyst. The MEA was then placed in a 5 cm^2 cell hardware provided by Fuel Cell Technologies inc. Then, the cell was connected to a test station for hydrogen fuel cells, which was used to control the gas flow at the anode and cathode and their relative humidity (RH). The system was conditioned by flowing fully humidified H_2 (99.999% Nippon gases) and N_2 (99.999% Nippon gases) at the cathodic and anodic sides, respectively, until the measured open circuit electric potential reached 0.3 V or less. Then, the cell was equilibrated by performing cyclic voltammetry ranging from 0.05 to 0.8 V with a sweep rate of 60 mV s^{-1} until reproducible and characteristic voltammograms were recorded. Finally, chronoamperometry at 0.35 V was performed until a stable current value was reached. Hydrogen permeability through a membrane correlates to the measured current according to Faraday's law[21].

Thermal characterization

Table S2. Glass transition temperatures (T_{gs}) and heat capacity variations (ΔC_p) for g-VBC-x-Q/PPOn and the corresponding g-VBC-x-Q membranes

**Membrane**	**T_{g1} [a] (°C)**	**ΔC_{p1} [b] (J (g·K)$^{-1}$)**	**T_{g2} [a] (°C)**	**ΔC_{p2} [b] (J (g·K)$^{-1}$)**
g-VBC-22-Q	-81	0.08	105	0.1
g-VBC-22-Q/PPO3	-82	0.06	102	0.2
g-VBC-22-Q/PPO5	-80	0.1	108	0.2
g-VBC-22-Q/PPO10	-74	0.2	107	0.2
g-VBC-27-Q	-84	0.06	105	0.1
g-VBC-27-Q/PPO3	-86	0.1	106	0.1
g-VBC-27-Q/PPO5	-78	0.1	107	0.1
g-VBC-32-Q	-89	0.01	104	0.1
g-VBC-32-Q/PPO3	-81	0.2	109	0.2
g-VBC-32-Q/PPO5	-80	0.2	110	0.1

[a] T_{g1} and T_{g2} values from the second heating cycle. [b] Heat capacity variation corresponding to glass transition temperature.

Table S3. Temperatures of maximum degradation (T_n), weight loss (Δw_n), and residue for g-VBC-x-Q/PPOn and the corresponding g-VBC-x-Q membranes

Membrane	T_1 (°C)	Δw_1 (%)	T_2 (°C)	Δw_2 (%)	*Residue* *(%)*
g-VBC-22-Q/PPO5	217	8.6	422	82.7	8.7
g-VBC-27-Q	220	12.1	423	86.7	1.2
g-VBC-27-Q/PPO5	222	12	426	80.5	7.5
g-VBC-32-Q	221	11.1	419	88.2	0.7
g-VBC-32-Q/PPO5	220	10.3	422	74.5	15.2

Mechanical tests

Table S4. Young's modulus (E), tensile strength (σ_{max}) and elongation at break (ε_b) of g-VBC-x-Q/PPOn membranes and relative g-VBC-x-Q copolymers. Films were conditioned overnight in 1 M $KHCO_3$ aqueous solution before being tested

Membrane	E (MPa)[a]	σ_{max} (MPa)	ε_b (%)
g-VBC-22-Q/PPO3	182 ± 37	4 ± 2	32 ± 13
g-VBC-22-Q/PPO5	195 ± 11	5 ± 2	37 ± 17
g-VBC-22-Q/PPO10	285 ± 29	7 ± 1	23 ± 9
g-VBC-27-Q	*62 ± 12*	*3 ± 1*	*70 ± 33*
g-VBC-27-Q/PPO3	90 ± 10	3 ± 1	68 ± 30
g-VBC-27-Q/PPO5	147 ± 12	4 ± 1	54 ± 41
g-VBC-32-Q	52 ± 7	3 ± 1	81 ± 21
g-VBC-32-Q/PPO3	90 ± 8	2 ± 1	54 ± 25
g-VBC-32-Q/PPO5	106 ± 18	3 ± 1	44 ± 18

[a] Calculated as the slope of the linear region.
